# Sectoral integration on an emerging stock market: a multi-scale approach

**DOI:** 10.1007/s11403-023-00383-y

**Published:** 2023-04-13

**Authors:** Kingstone Nyakurukwa, Yudhvir Seetharam

**Affiliations:** School of Economics and Finance, University of the Witwatersrand, Johannesburg, South Africa

**Keywords:** South Africa, Sectoral contagion, Connectedness, Spillovers, Integration, C58, G01, G11

## Abstract

The purpose of this study is to examine the connectedness of industry sectors on the Johannesburg Stock Exchange in a time–frequency domain. We use econophysics-based methods like the wavelet multiple correlation and wavelet scalogram difference to identify the evolution of the connectedness of the sectors over time and at different frequencies. The findings show that the sectors on the Johannesburg Stock Exchange are especially integrated at lower frequencies. Wavelet multiple correlation peaks in response to local and global shocks like the black-swan COVID-19 pandemic in 2020 and the downgrading of South African debt by Fitch in 2013. Though there are opportunities for sectoral diversification on the JSE, this fails when it is most needed, during crisis periods. Investors should therefore consider other asset classes that could serve as a haven in times of crisis. Though extant literature has examined sectoral dependencies on the stock markets of developed and developing countries, to the best of our knowledge, this is the first study to examine this connectedness in a South African context using multiple nonparametric methods that are robust to non-normality, presence of outliers as well as non-stationary data.

## Introduction

One of the fundamental tenets of classical finance is the need for portfolio diversification to reduce the risk of a portfolio (Markowitz 1952). Diversification of risk can be implemented at different angles and different levels, including at the stock level by picking unrelated stocks (Zaimovic et al. 2021), at the sectoral level by investing in weakly correlated sectors (Carrieri et al. 2012), as well as at the international level by picking stocks from countries that are weakly integrated (Shawky et al. 1997). A country like South Africa is often considered the gateway into Africa and is used by different investors, national and international, for the purposes of portfolio diversification (Nyakurukwa 2021). It is therefore essential to understand the extent to which sector indices in the South African context are connected as well as the dynamics of risk spillover across the sectors to uncover the possibilities of sectoral diversification in such an important market.

According to Wu et al. (2019), since the 2008 Global Financial Crisis, there have been several global events that have resulted in financial markets across the globe exhibiting contagious patterns. These global events include the European debt crisis, Brexit and the COVID-19 pandemic (Bello et al. 2022). These global events have increased the propensity for risks to spread across countries, markets and sectors with potentially adverse effects on capital markets. It is therefore of paramount importance to comprehend the dynamics of how risks are transmitted across financial markets. Wu et al. (2019) posit that while several studies have explored the interconnectedness of financial markets using different methodologies, the focus has shifted to the identification of the source of interconnectedness in recent times.

The introduction of the notion of Systematically Important Financial Institutions (SIFIs) in 2010 (in reaction to the effects of the Global Financial Crisis of 2008) went a long way in establishing regulatory frameworks to identify the specific institutions that can contribute significantly to disturbances in the wider financial system. The contribution of specific institutions to the failure of the wider financial system can be due to their size, leverage and other factors. While the original notion of SIFIs is specific to financial institutions, the principles can be applied to sectors or markets (Wu et al. 2019). Ranjeeni (2014) argues that due to heterogeneities in risk characteristics, it is possible that shocks to one sector can be transmitted to other sectors. The determination of leading industries and sectors helps in the comprehension of the transmission of risks across sectors, and this is useful, especially in risk management and portfolio diversification. An understanding of the patterns of contagion across sectors is also useful for policymakers as well as regulators as this helps them draft tailor-made policies in unison with specific risk patterns.

South Africa has been chosen ahead of other emerging and frontier markets in Africa for several reasons. The country has the deepest and widest financial system on the continent and has often been used as a gateway into Africa by many international and institutional investors. With a market capitalisation of more than $US1 trillion and ranking in the top twenty^1^ bourses by market capitalisation, South Africa provides real possibilities for sectoral diversification from an African perspective. Second, South Africa is different from other emerging markets like China, for example, as it is dominated by institutional investors while the latter is dominated by retail investors. It is usually institutional investors who have the resources and the technical capacity to explore sectoral diversification and thereby making this study important in such an environment.

Additionally, according to Malik (2021), the past two decades have seen an increased propensity for sector investing because of the popularity of Exchange Traded Funds (ETFs). From an African perspective, South Africa offers the widest and deepest ETF market, having its first ETF listed way back in 2000, providing a *raison d'être* for a study of this nature. Though it is expected that in the long run, the relationship among sectors is strongly positively correlated, there can be substantial fluctuations in the short run. An understanding of the sectoral dependence on the JSE in a time–frequency domain, therefore, provides new insights away from the other emerging as well as developed markets.

Empirically, several studies have been conducted on the aggregate comovement of sector returns using various methods. The majority of the studies that have been done on return and volatility connectedness have been mainly concentrated on developed markets. Hassan and Malik (2007) examined the transmission of volatility shocks among six sectors in the USA. The authors report significant transmission of volatility shocks among the sectors using multivariate GARCH models. The authors also conclude that there is cross-market hedging by investors in these markets. Yue et al. (2020) undertook a comparative analysis of information transfer among the stock markets of China and the USA using transfer entropy. The authors report that the industry sectors in the two countries are more synchronised during turmoil periods. Noulas et al. (2021) estimate a dynamic equicorrelation multivariate GARCH model (DECO-MGARCH) and time-dependent entropic measures on the sector returns of the Athens Stock Exchange and report that investors are likely to share the same opinions during crises and thereby leading to high sectoral correlations during these periods.

Several of the recent studies on sectoral connectedness studies have been done in the context of the COVID-19 pandemic to uncover how the connectedness of stock market sectors react to black-swan events. Shahzad et al. (2021a, b) examine asymmetries in realised volatility connectedness among Chinese sectors using 1-min data between 2019 and 2020. The study gives evidence of the asymmetric effect of good and bad volatilities, and this effect is amplified during the COVID-19 period. Using US-equity sectors, Shahzad et al. (2021a, b) show that networks of generalised forecast error variance decomposition underwent an interesting rearrangement during the pandemic period as dominant clusters grew closer, while the rest of the network remained widely spaced. Hernandez et al. (2022) examine return spillovers under high/low volatility regimes across US sectors and report that energy is the largest transmitter and receiver of spillovers to/from other sectors. Additionally, spillovers intensify following the occurrence of the COVID-19 pandemic.

Few studies on the connectedness of sectoral returns have also been done in developing countries. In South Africa, Katzke (2013) utilised the Dynamic Conditional Correlation (DCC) and Asymmetric-DCC Multivariate Generalized Autoregressive Conditional Heteroskedasticity (MV-GARCH) techniques to isolate the time-varying conditional correlations from the conditional variance component on JSE sector indices. The author used the following sectors; financials, industrials, consumer goods, consumer services, telecommunications and basic materials. Katzke (2013) reports results that suggest that the use of static models to examine the interconnectedness of stock market sectors is likely to be misspecified as the comovement evolves over time. The author also reports results that suggest that periods of local and global uncertainty amplify comovements between sectors and thereby weakening diversification efforts across sectors. In Pakistan, Aslam et al. (2022) show that the stock market is driven by the oil and gas marketing sector, as well as the oil and gas exploration sector, with heavy dependence on commercial banks, fertilisers, power generation and distribution and automobile parts and accessories industries.

We seek to expand the empirical studies outlined above by investigating the sectoral return comovement in a time–frequency domain. Though the efficiency market hypothesis (EMH) assumes that investors are homogeneous, the fractal market hypothesis (Peters 1994) and the heterogeneous markets hypothesis (Müller et al. 1995) emphasise the heterogeneity of investors based on their different investment horizons. Some investors invest in the short term (e.g. naïve investors), medium term (e.g. hedge funds) and long term (e.g. pension funds). The reaction of these different types of investors to external shocks is likely to be heterogeneous because of the differences in the risk profiles of the investors. It is therefore essential to examine this relationship in a time–frequency domain. Though there are studies that have attempted to examine sectoral dependence using multi-scale approaches, these have tended to concentrate on developed markets, utilising different asset classes (e.g. Hamdi et al. 2019) and mostly examining cross-country sectoral connectedness (e.g. Hanif et al. 2021).

Methodologically, we use a wider range of methods from econophysics to understand the sectoral connectedness of sector indices in South Africa. The increased use of econophysics in the analysis of financial data is providing new insights into stock market dynamics. Econophysics treats financial markets as complex systems comprised of multiple subsystems and networks (Zaheer et al. 2022). These multiple subsystems include among others, different investors, different asset classes, different stock market sectors and different types of investors. The interactions and information flow among the different subsystems are crucial to fully understanding the dynamics of the system as a whole (Yue et al. 2020). We explore the interactions in one of the subsystems; sector indices on the Johannesburg Stock Exchange, to understand the whole complex system of the exchange using methods borrowed from statistical mechanics and theoretical physics.

Physicists have contributed immensely to the modelling of complex systems, and the application of these methods to financial and economic systems has given rise to a whole new analytic domain, econophysics (Nguyen and He 2015). The interaction of multiple agents in the stock market, often with different objectives and expectations, leads to financial data that are not stationary and justifies the need to assess the dynamics of the stock market at different horizons to cater for different investors (In and Kim 2012). Methods from econophysics, therefore, become handy for the robust analysis of financial time series. Methods from econophysics are also robust in the presence of the widely reported stylised facts of financial variables like excess kurtosis, leptokurtic distributions, volatility clustering, structural breaks and many others (Nguyen and He 2015). Our sample period includes a black-swan event (COVID-19), which notably led to regime shifts and structural breaks in financial variables and therefore justifying the need for the adoption of methods that are robust in the presence of these stylised facts.

The findings from the study show that correlations are higher as the frequency considered decreases. We also report that multiple correlations are at their highest in response to significant local and global events. This is seen from the spike in multiple correlations after the announcement of the national lockdown measures introduced in 2020 to contain the spread of positive COVID-19 cases. A spike in wavelet multiple correlations is also observed when the country’s long-term foreign and local currency debt ratings were downgraded by Fitch. Though the results show increased wavelet multiple correlations at the lowest frequencies, there is still room for sectoral portfolio diversification on the JSE as shown by the dynamic nature of sectoral index similarity with the benchmark index. The wavelet multiple correlation results show that there is no clear leader or follower at higher frequencies, while at lower frequencies, the financials, as well as the industrials, are seen as potential followers in the connected system. This shows that in the long term, these two sectors are significantly affected by what happens in the other sectors. The results are in line with most of the mainline literature on the connectedness of financial markets, showing that connectedness is more pronounced at lower frequencies and is influenced by global and local significant events (Katzke 2013; Noulas et al. 2021; Yue et al. 2020).

The study proceeds as follows: in the next section, we outline the methods and materials used in the study, followed by the presentation of the results and the discussion thereof. We end with the conclusion that wraps up the study and provides policy implications of the study.

## Methods and materials

### Data

The Johannesburg Stock Exchange uses the Industry Standard Benchmark (ICB) for categorising and classifying companies into various constituent indices. ICB is globally recognised and is widely used by different stakeholders and institutions including the London Stock Exchange and the New York Stock Exchange. The classification of shares and securities is divided into four levels. These levels are Industrial (Level 1), supersector (Level 2), Sector (Level 3) and Subsector (Level 4). The focus of this study is on level 1 which has 11 industries^2^ as shown in Table 1.

**Table 1:** Classification of indices on the JSE

	Index code	Index name	ICB industry code
1	JI0010	Technology	10
2	JI0015	Telecommunications	15
3	JI0020	Healthcare	20
4	JI0030	Financials	30
5	JI0035	Real estate*	35
6	JI0040	Consumer discretionary	40
7	JI0045	Consumer staples	45
8	JI0050	Industrials	50
9	JI0055	Basic materials	55
10	JI0060	Energy*	60
11	JI0065	Utilities*	65

The sectors on the JSE and the respective ICB industry codes. The sectors marked in asterisks are not included in the study because of fewer data points. We use the index codes to represent the different sectors in the rest of the study

From the 11 industries in level 1 shown in Table 1, we exclude 3 sectors, namely JI0035, JI0060 and JI0065, since they were introduced later and therefore have fewer data points. This leaves us with 8 industries. We use J203 (All Share Index) as the benchmark index. Sectoral returns are calculated as follows:

1 $$ r = \log \left( {\left. {\frac{{P_{t} }}{{P_{{}} }}} \right)} \right. $$

where $r$ is the return at time $t$; ${P}_{t}$ is the closing price of a sectoral index at time $t$ and ${P}_{t-1}$ is the closing price of a sectoral index at time $t-1$. The sample period starts from 1 January 2005 to 31 December 2021 (the last available date when data were collected). The starting date of the sample is chosen because JSE only started incorporating ICB classifications in 2005. The analysis is done at daily granularity. The final dataset consists of 8 sector indices and the benchmark index with 3945 daily observations and which excludes weekends and public holidays where there is no trading.

### Empirical methods

In this section, we outline the empirical methods that we deploy to understand the extent of sectoral return connectedness on the JSE.

#### Bivariate wavelet correlation

We start by examining the nature of the time–frequency connectedness of JSE sectoral returns at a bivariate level. This provides an understanding of how each sector comoves with all other sectors independently in a time–frequency space. To find the pairwise correlation between sectors at multiple frequencies, we utilise bivariate wavelet correlation. The process of computing the wavelet correlation is attained by creating variances between two time series at various identified wavelet scales. According to Tiwari et al. (2013), wavelet variance can be defined as the substitution of variability over specific scales for the global measure of variability estimated by sample variance. Given a stochastic process X, the wavelet covariance is approximated using the maximal overlap discrete wavelet transform (MODWT) for scales ${\tau }_{j}={2}^{j-1}$ through:

2 $$\hat{\sigma }_{x}^{2} \left( {\tau _{j} } \right) = \frac{1}{{\hat{N}_{j} }}\sum\limits_{{k = L_{j}  - 1}}^{{N - 1}} {\left( {\widehat{W}_{{j,k}} } \right)^{2} } ,$$

where ${\widehat{W}}_{j,k}$ is the MODWT wavelet coefficient of variable *X* at scale ${\tau }_{j}$, ${\widehat{N}}_{j}$ is the number of coefficients that are unaffected by the boundary, ${L}_{j}$ is the length of the scale ${\tau }_{j}$ wavelet filter.

The decomposition of the covariances between pairs of stochastic processes is done on a scale-by-scale basis. The wavelet covariance at scale ${\tau }_{j}$ is represented by:

3 $$ \gamma_{XY} \left( {\tau_{j} } \right) = cov_{XY} \left( {\tau_{j} } \right) = \frac{1}{{\hat{N}_{j} }}\mathop \sum \limits_{{k = L_{j} - 1}}^{N - 1} \hat{W}_{j,k}^{x} \hat{W}_{j,k}^{y} , $$

With a wavelet covariance for ($x_{t} , y_{t}$) and wavelet variances for ($x_{t}$) and ($y_{t}$), the MODWT approximation of the wavelet correlation is given by:

4 $$ \hat{\rho }_{xy} \left( {\tau_{j} } \right) = \frac{{{\text{Cov}}_{xy} \left( {\tau_{j} } \right)}}{{\hat{\sigma }_{x}^{2} \left( {\tau_{j} } \right)\hat{\sigma }_{y}^{2} \left( {\tau_{j} } \right)}}, $$

#### Wavelet multiple correlation (WMC)

The bivariate wavelet correlation described above has been criticised because of several limitations, and the wavelet multiple correlation suggested by Fernández-Macho (2012) has often been proffered as more succinct. First, the traditional pairwise wavelet approaches that utilise $n$ variables to estimate correlation and cross relation lead to excessively high pairwise correlation coefficients $(n(n-1)/2)$, which may be difficult to accurately interpret. Also, Timari et al. (2013) emphasise the weakness of pairwise wavelet coefficients in a multivariate framework as possible relationships among the variables may lead to spurious coefficients. These and other weaknesses make wavelet multiple correlation superior and clearer to interpret than the pairwise coefficients. Given that a multivariate stochastic process is given by.

${X}_{t}=({x}_{1t}, {x}_{2t}, \dots , {x}_{mt})$ and that respective scale ${\lambda }_{j}$ is represented by ${W}_{jt}={w}_{1jt, }{w}_{2jt},\dots , {w}_{njt})$, the wavelet coefficients can be estimated by applying MODWT to each ${x}_{it}$ process. The WMC is estimated as follows:

5 $$ \varphi_{X} \left( {\lambda_{j} } \right) = \sqrt {1 - \frac{1}{{\max {\text{diag}} P_{j}^{ - 1} }},} $$

where ${P}_{j}$ refers to the $n\times n$ correlation matrix of ${W}_{jt}$ and the max diag (.) operator provides a selection for the largest element in the diagonal of the argument.

#### Wavelet multiple cross-correlation

To identify a potential leader that can influence other indices among the stock indices, we utilise the wavelet multiple cross-correlation (WMCC). By allowing lag $\tau$ between the observed and the fitted values of the criterion variable at each scale ${\lambda }_{j}$, the WMCC is defined as

6 $$ \varphi_{X,t} \left( {\lambda_{j} } \right) = {\text{Corr}}\left( {w_{ijt} \hat{w}_{ijt + \tau } } \right) = \frac{{{\text{Cov}}\left( {w_{ijt} , \hat{w}_{ijt + \tau } } \right)}}{{{\text{Var}}\left( {w_{ijt} } \right){\text{Var}}\left( {\hat{w}_{ijt + \tau } } \right)}} $$

where ${w}_{ijt}$ on the set of regressors $\left\{{w}_{kj}, k\ne j\right\}$ leads to the maximisation of the coefficient of determination ${\varphi }_{X}\left({\lambda }_{j}\right)$ and ${\widehat{w}}_{ij}$ is the fitted value of the regression.

#### Wavelet scalogram difference

We use the concept of the wavelet scalogram difference (WSD) to identify the periods and frequencies in which two time series are significantly similar or dissimilar. This concept was introduced by Bolós et al. (2017) to compare the behaviour of two time series through their respective scalograms for different windows in time and scale, thus allowing to ascertain the particular scales and time intervals in which both time series exhibit similar pattern, comparing their scalograms and determining whether they give the same weight to the different scales. According to Bolós et al. (2017), the WSD can detect features that go unnoticed in the most used wavelet squared coherence. We compare all the industry sectors vis-à-vis the benchmark index to establish where the indices are most similar in a time–frequency domain.

Using the wavelet scalogram difference concept, time series with similar behaviour have similar windowed scalograms (WS) resulting in values that are close to zero after ${\mathrm{log}}_{2}({WSD}^{-1})$ transformation. The ${\mathrm{log}}_{2}({WSD}^{-1})$ transformation is particularly applied to create a scale comparable to the WSD. Given two time series $x$ and $y$, the WSD is computed centred at time $t$ and log-scale $k$ where $k\in {\mathbb{R}}$ for the continuous wavelet transformation (CWT) with log-scale radius $r$ and time radius $\tau$ as follows:

7 $$ WSD_{\tau ,r} \left( {t,k} \right) = \left( {\mathop \int \limits_{k - r}^{k + r} \left( {\frac{{WS_{\tau } \left( {t,k} \right) - WS_{\tau }^{^{\prime}} \left( {t,k} \right)}}{{WS_{\tau } \left( {t,k} \right)}}} \right)^{2} {\text{d}}k} \right)^{\frac{1}{2}} $$

where ${WS}_{\tau }$ is the WS of time series $x$ and ${WS}_{\tau }^{^{\prime}}$ is the WS of time series $y$. The details of how the method deals with boundary effects are described by Bolós et al. (2017). The concept of WSD has since been used in various empirical studies (such as Raath and Ensor 2020).

## Results and discussion

### Descriptive statistics

**Fig. 1:**
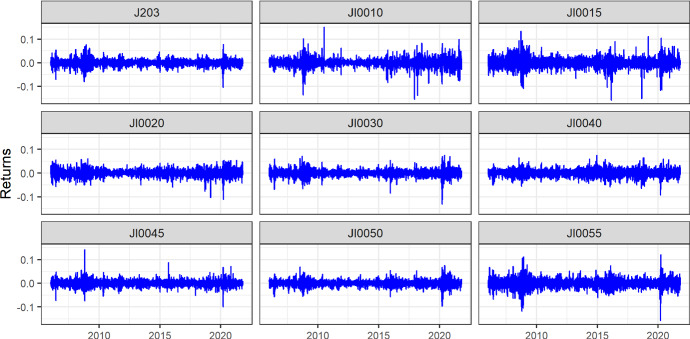
Return series of the sectors

This section presents the descriptive statistics of the time-series variables used in this study. The closing prices of the sector indices as well as the accompanying return series are visualised in Fig. 8 (Appendix) and Fig. 1, respectively.

Figure 1 demonstrates the stylised fact common in financial time series, volatility clustering. The biggest drops in sectoral returns are observed in 2008 and 2020, two periods coinciding with the Global Financial Crisis and the COVID-19 pandemic. Also evident in Fig. 1 is the effect of the pronouncement of COVID-19 as a global pandemic in March 2020, which led to crashes in all the sectoral indices. Table 2 shows the mean, standard deviation, Jarque–Bera (JB) statistics as well as Augmented Dickey–Fuller test statistics (ADF).

**Table 2:** Descriptive statistics, normality and stationarity tests

Index code	Mean	S.D	JB	ADF
JI0010	0.00035	0.01673	15,459***	− 15.311***
JI0015	0.00017	0.02010	5822.8***	− 17.441***
JI0020	0.00037	0.01386	2642***	− 15.945***
JI0030	0.00019	0.01372	10,842***	− 16.169***
JI0040	0.00063	0.01432	726.79***	− 15.561***
JI0045	0.00050	0.01267	9152.7***	− 16.193***
JI0050	0.00020	0.01241	3387.3***	− 15.141***
JI0055	0.00026	0.01885	4549.1***	− 15.656***
J203	0.00034	0.01343	3986.6***	− 15.975***

The descriptive statistics of the sectoral returns. The index code shows the different sectors sampled as defined in Table 1. S.D, JB and ADF represent the standard deviation, Jarque–Bera test statistics and augmented Dickey–Fuller test, respectively. *** shows statistical significance at the 1% level

**Fig. 2:**
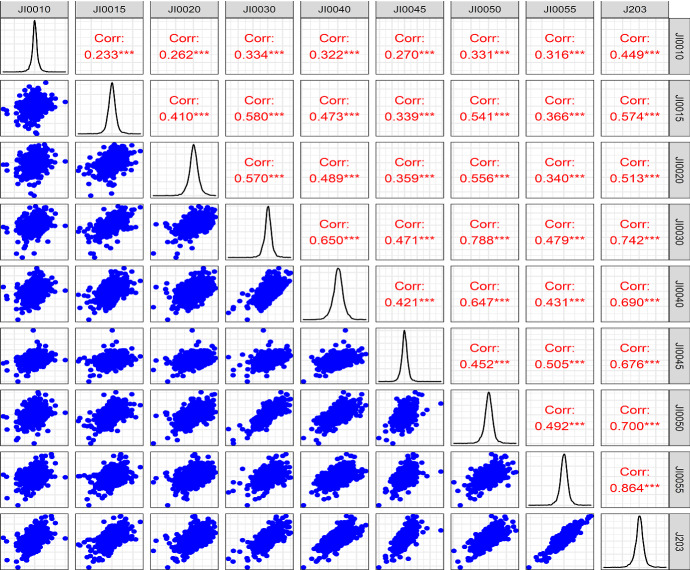
Pairwise correlation structure of the variables. The graph shows the pairwise correlation among the sectors, scatter plots of the sectoral returns as well as distributions of returns. *** represents a correlation coefficient that is statistically different from zero. This was visualised using the GGally R package (Schloerke et al. 2021)

Table 2 shows that JI0040 has the highest daily mean return, while the lowest daily mean return was recorded for JI0015. In terms of the standard deviation (S.D), the most volatile sector is JI0015, while the least volatile sector is JI0050. All the series are not normally distributed as demonstrated by the Jarque–Bera (JB) test statistics, which are all statistically significant at the 1% level of significance, thereby rejecting the null hypothesis of normality. The augmented Dickey–Fuller (ADF) test statistics for all the sectors are statistically significant at the 1% level of significance. This leads us to reject the null hypothesis of a unit root in the series, and it can therefore be concluded that the sector series are all stationary. The fact that the series are not normally distributed requires the utilisation of nonparametric econometric methods. Thus, the use of econophysics ameliorates this situation as the methods are nonparametric and are efficient in the presence of structural breaks and regime shifts. The Pearson pairwise correlation relationships between sectors are visualised in Fig. 2.

Figure 2 shows the pairwise correlations between the sectoral indices. All the pairwise relationships are statistically significant at the 1% level. The highest pairwise Pearson correlation is between J203 and JI0055 (*r* = 0.864), and the lowest pairwise correlation coefficient is between JI0010 and JI0015 (*r* = 0.233). It can also be noted that most of the pairwise coefficients are greater than 0.5, showing close positive relationships between the sampled sectors. There is therefore prima facie evidence of potential return contagion and spillovers, which should be investigated using more robust econometric models. Figure 2 also shows the presence of outliers (from the scatter plots) in several of the time series, thereby reducing the efficacy of traditional parametric methods in examining the relationships among the sectors.

### Wavelet bivariate correlation

**Fig. 3:**
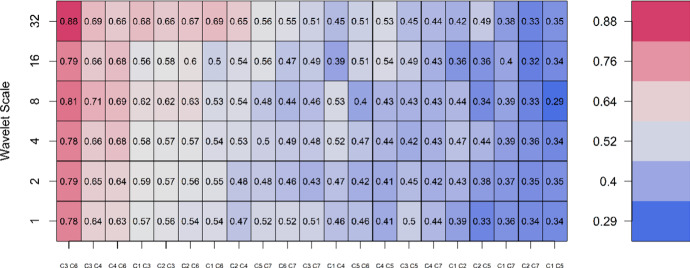
Wavelet bivariate correlation. The colour scale ranges from dark blue (low correlations) to dark red (high correlations). We examine the pairwise relationships up to a wavelet scale of 32 to ameliorate edge effects as well as spurious relationships at lower frequencies. We used the codes CI, C2, C3, C4, C5, C6, and C7 (Analysis software could not allow us to customise) to represent the following sectors, respectively; JI0015, JI0020, JI0030, JI0040, JI0045, JI0050, JI0055 as defined in Table 1

We first report the wavelet bivariate correlations among the sectors to establish the relationships in a frequency space. Figure 3 visualises the pairwise relationships among the sectors sampled in the study. We decompose the sectoral return series into seven details ${w}_{i1\dots }$ ${w}_{i7}$). The details of the interpretation of the dynamics of each wavelet coefficient are shown in Table 3.

**Table 3:** Interpretation of the wavelet scales

${w}_{i1}$	2–4 days	Intraweek scale
${w}_{i2}$	4–8 days	Weekly scale
${w}_{i3}$	8–16 days	Fortnightly scale
${w}_{i4}$	16–32 days	Monthly scale
${w}_{i5}$	32–64 days	Monthly to quarterly scale
${w}_{i6}$	64–128 days	Quarterly to biannual scale
${w}_{i7}$	128–256 days	Biannual to annual scale

How the different wavelet scales are interpreted where the details from ${w}_{i1}$ to ${w}_{i7}$ represent the following scale levels; 1, 2, 4, 8, 16, 32, 64

In Fig. 3, the most closely related sectors are to the left of the Figure and the sectors become less closely related as we move to the right of the Figure. The most correlated sectors across the frequency spectrum are the JI0030 (C1) and JI0050 (C6) followed by the relationship between JI0030 (C3) and JI0040 (C4). The fact that JI0030 appears several times to the left of Fig. 3 (showing its strong positive relationships with other sectors) shows the importance of this sector in the connected system. This corroborates the study by Small and Hsieh (2017) who reported that on the Johannesburg Stock Exchange, financial stocks are exposed to significant value risk and to an extent influenced by the performance of large stocks.

### Wavelet multiple correlation

In this section, we report the results from the wavelet multiple correlation using the 8 sectors sampled for the study. Within a multivariate context, this gives a succinct way of visualising multiscale correlations compared to the pairwise correlations reported in the previous section. Tiwari et al. (2013) point out the advantages of using wavelet multiple correlation in a multivariate framework that include protection against Type 1 errors as well as protection against spurious correlations.

**Fig. 4:**
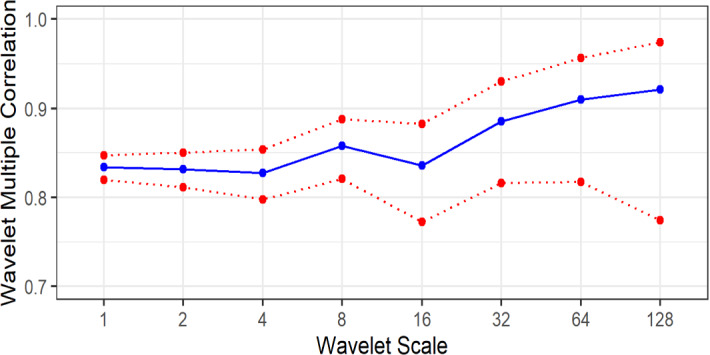
Wavelet multiple correlation. The wavelet multiple correlations (blue continuous line) at the different scales as defined in Table 3. The red dotted lines show the 95% confidence bands of the wavelet coefficients

Figure 4 displays the wavelet multiple correlation coefficients, and the corresponding 95% confidence bounds from the returns of the 8 industries sampled for this study.

**Fig. 5:**
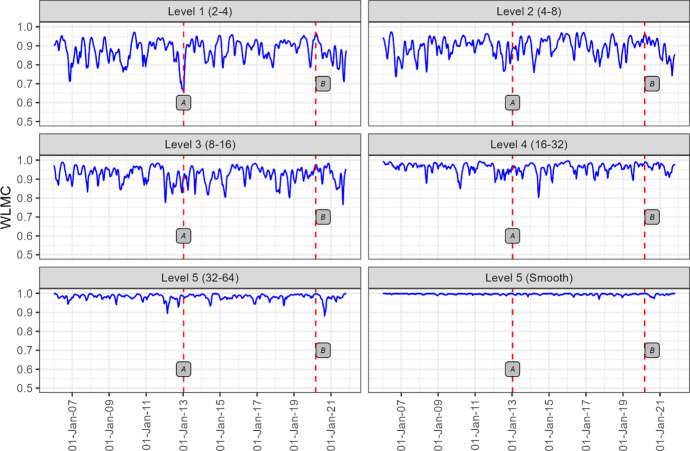
Wavelet multiple correlation in the time–frequency domain. The WLMC at 5 different levels, where each level represents different investing horizons shown in brackets. Some important events in South Africa are demarcated by red vertical dotted lines labelled A, B and C. The label “A” corresponds to 10 January 2013 when Fitch Group downgraded South Africa's long-term foreign currency Issuer Default Rating to 'BBB' from 'BBB + ' and long-term local currency IDR to BBB + from A. The label “B” corresponds to 11 March 2020 when COVID-19 was declared a global pandemic

Figure 4 shows that the wavelet multiple correlation coefficients are high, with the highest frequency having a coefficient of 0.83 and the lowest frequency with a coefficient of 0.91. The correlation coefficients are therefore high at all the frequencies and are inversely related to frequency intervals. At the lowest frequency, though the wavelet multiple correlation is at its highest, there is still room for portfolio diversification as the sectors are not perfectly correlated. Figure 4 shows how the aggregate comovement of the sectors evolves in frequency but does not show the evolution of the comovement in time. We visualise the evolution of the multiple correlations of the sectors both in time and frequency in Fig. 5. Figure 5 shows the heterogeneity of the correlation structure of these sectors over time and across frequencies. It is also evident that above the monthly scale, the long-term correlation structure is stable with multiple correlations close to 1 at the longest time scale. This implies that at the quarterly scale, returns obtained in any one of the sectors used in this study can be determined by the overall performance in other industries.

**Fig. 6:**
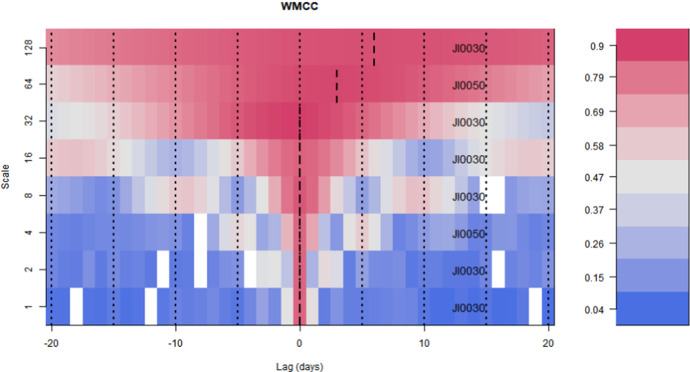
Wavelet multiple cross-correlation^3^ The wavelet multiple cross-correlations among 8 industries' market returns. The wavelet coefficients are within the 95 percent confidence interval for each wavelet correlation. Zones in which the 95 percent interval spans zero are indicated in white. The long-dashed vertical lines indicate where in time the strongest wavelet correlation values are localised

Figure 5 shows that at the highest frequency, there is an increase in multiple correlation coefficients resulting from local and global events. At lower frequencies, however, there is no clear pattern that shows how multiple correlations react to local and global events labelled in Fig. 5. It can therefore be concluded that multiple correlations increase when reacting to negative local and global events at higher-frequency intervals than at lower-frequency intervals. The rise in multiple correlations around negative events gives evidence of financial contagion across the sectors. The fact that multiple correlations spike at higher-frequency intervals could be a result of overreaction and herding by retail investors who normally have short investment horizons. To identify the sectors that have the potential to lead or lag other sectors at different wavelet scales, we utilise the wavelet multiple cross-correlation results, which are visualised in Fig. 6.

Figure 6 shows the wavelet multiple cross-correlations among 8 industries' market returns. The wavelet coefficients are within the 95 percent confidence interval for each wavelet correlation. Zones in which the 95 percent interval spans zero are indicated in white. The long-dashed vertical lines indicate where in time the strongest wavelet correlation values are localised.

Figure 6 shows the multiple cross-correlations for the various wavelet scales consistent with leads and lags of up to 20 trading days. The sector that maximises the wavelet multiple correlations against the linear combination of other industries is shown on the right-hand side of the Figure. Also, in Fig. 6, the black dashed line shows the time lag at which the strongest wavelet correlation coefficients are localised. It is evident from Fig. 6 that there are no return spillovers between the 2–4 day and 32–64-day frequencies since localisations occur at the point of symmetry (i.e. zero time lag). There are significant return spillovers at the highest scales of 64 and 128 corresponding to the 64–128 and 128–256-day frequencies, respectively. In Fig. 6, positive time lags are interpreted as lagging sectors while negative time lags are indicative of leading industries. JI0050 lags within the 64–128 days while JI0050 lags in the 128–256-day frequency.

### Wavelet scalogram difference

**Fig. 7:**
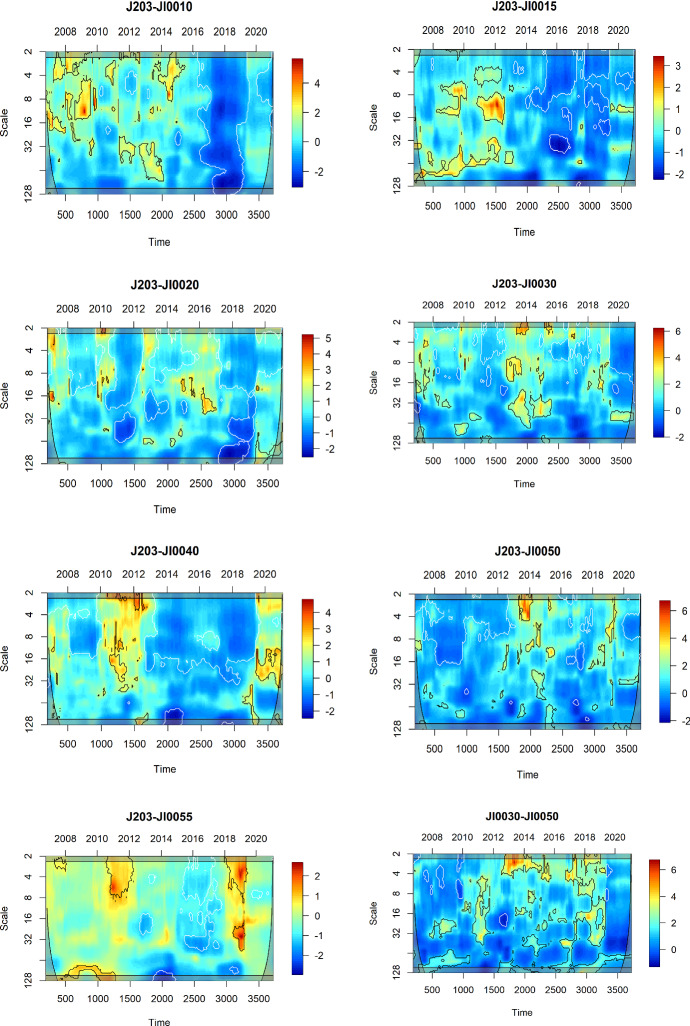
Wavelet scalogram difference. The white-line regions are regions of statistical significance at the 5% level (high similarity), and the black-line regions are regions of statistical significance at the 95% level (low similarity) estimated using Monte Carlo simulations. The box and thin black region are the border effects and define the cone of influence

**Fig. 8:**
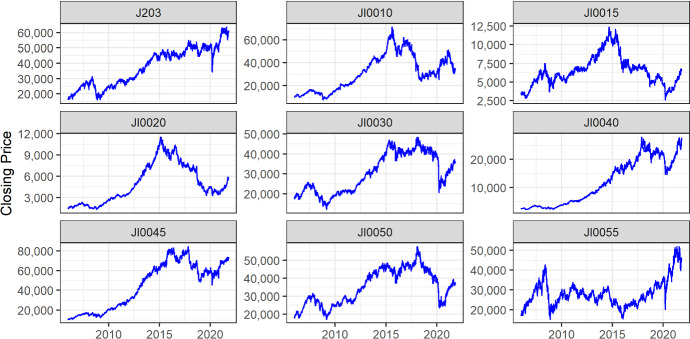
Closing prices of the indices. The visualisation of the closing price series of the sampled JSE sectors

Figure 7 shows the wavelet scalogram difference diagram for all the sectors vis-à-vis the benchmark index to establish if they follow similar patterns by comparing their respective scalograms for different windows in time and scale. The wavelet scalogram difference for JI0030 and JI0050 is also included to establish how these two sectors are related in the time–frequency domain, given that the results from the wavelet multiple correlation showed that these two sectors have the potential to lag other sectors.

In Fig. 7, the white-line regions are regions of statistical significance at the 5% level (high similarity) and the black-line regions are regions of statistical significance at the 95% level (low similarity) estimated using Monte Carlo simulations. The space between the box and thin black border lines show the border effects and define the cone of influence.

Though Fig. 7 shows intermittent phases of dissimilarities between the benchmark index and the sampled indices, for almost all the sectors, similarity is concentrated and statistically significant around the year 2018. The similarity between the benchmark index and the sectoral indices around this time is significant especially at higher frequencies, with other sectoral indices having significant similarities even across the whole spectrum of the frequency domain (e.g. J203-JI0010). This shows that prospects of benefiting from sectoral diversification existed on the Johannesburg Stock Exchange outside the year 2018 as there was room for diversification. In South Africa, 2018 was an eventful year that was characterised by several political events that changed the sentiment of investors on the stock market. In 2018, South Africa experienced its first recession since the Global Financial Crisis of 2008 (Thukwana and Mosteiro 2018). In the same year, after years of talks about state capture in South Africa, the then President of the republic announced a commission of enquiry into state capture. However, the president did not see the work of the commission to completion as he later officially resigned in the same year upon being implicated. All these events affected the stock market as most media outlets reported a rally on the Johannesburg Stock Exchange across all the sectors as the investors expected the “business-friendly”^4^ new president to instil confidence back into the financial markets.^5^ Another period that exhibits phases of significant similarity between the benchmark index and most of the sectoral indices is the year 2020. This coincides with the declaration of COVID-19 as a global pandemic and the imposition of a national lockdown by the South African government to contain the disease. Since the COVID-19 pandemic was a black-swan event, no one knew how the disease would evolve and this uncertainty induced fear in financial markets leading to the crashing of the stock market across all sectors.

Generally, our results are in line with existing literature on the connectedness among sector returns in other markets. The time-varying connectedness of sector indices established in this study corroborate the results that have been reported in a South African context using a different methodological approach (Katzke 2013). The findings also show that sectoral connectedness is amplified in times of financial turmoil, consistent with existing literature (e.g. Noulas et al. 2021; Yue et al. 2020), demonstrating that sectoral diversification fails when it is needed the most. From a theoretical perspective, the assumption of investor homogeneity as hypothesised by the Efficient Markets Hypothesis (Fama 1965; Malkiel and Fama 1970) may not adequately explain the behaviour of investors in the South African market. The evidence of the heterogeneous evolution of sectoral connectedness on the Johannesburg Stock Exchange in time and across different frequencies suggests an inclination of the financial markets towards alternatives to the EMH like the heterogeneous markets hypothesis (Müller et al. 1995) and the Fractal Markets Hypothesis (Peters 1994).

## Conclusion

The purpose of this study was to examine the connectedness of JSE investible sectors in a time–frequency domain to establish the evolution of the relationship in time and across different frequencies. Using wavelet multiple correlation, the results of the study show that the coefficients are almost stable from wavelet scales 1–16 and thereafter increase gradually until the last scale of 128. This signals the predominance of homogenous investors in the intraweek, weekly, and fortnightly scales. Heterogeneity in investor characteristics can be observed from monthly frequencies going up. The results also show that wavelet multiple correlations increase whenever there are local and globally significant events. As a result, diversification across sectors is less beneficial around crises.

The results reported herein have several implications for investing practitioners as well as regulators. To investors who use sectoral diversification (e.g. ETFs), this form of diversification fails in times of crisis and as such portions of funds should be devoted to other alternative asset classes to avoid heavy losses during these times. For regulators, the results show that the financial sector has the potential to lag other sectors at lower frequencies, signifying that it is affected by other sectors. There is therefore a need to ensure that financial institutions are well capitalised and can stem any shocks from other sectors so that the shocks cannot cascade to the wider economy as the financial sector is crucial to the functioning of the whole economic system. Future studies could compare the connectedness of South African sectors with other sectors in developed and emerging markets. Since the results have established that the connectedness of the sectors on the JSE varies in a time–frequency space, future studies could also conduct a formal empirical test of the heterogeneous markets hypothesis as well as the Fractal Markets Hypothesis in a South African context. This will establish the nature of market efficiency on the JSE and therefore help investors to systematically identify mispriced assets in the market. Finally, future studies could also explore the sectoral dependence on the JSE while controlling for other macroeconomic variables like the exchange rate, the inflation rate and the interest rates. This can be achieved, for example, by utilising partial wavelet analysis to examine how sectoral returns comove while holding these macroeconomic variables constant. A notable limitation of the study is the exclusion of three sectors (real estate, energy and utilities) from the study because of few data points, which may mask the whole picture of sectoral connectedness on the JSE.

## Appendix

See Fig. 8.

## Footnotes

1 Bonga-Bonga and Rangoanana (2022).

2 In this study, we refer to the industries as sectors to avoid confusion with the “industrials” industry.

3 This was visualised using the “W2CWM2C” R Package (Polanco-Martínez & Fernández-Macho 2014).

4 https://www.reuters.com/article/emerging-markets-idUSL8N1Q528N.

5 https://www.bloomberg.com/news/articles/2018-02-15/zuma-s-exit-spurs-surge-in-south-african-stocks-as-banks-rally.
